# Identifying Candidate Mediators Linking ADHD Symptoms and Internalising Problems in Adolescence: An Exploratory Longitudinal Mediation Analysis

**DOI:** 10.1177/10870547261419589

**Published:** 2026-03-06

**Authors:** Aja Louise Murray, Katie Dryburgh, Edmund J. S. Sonuga-Barke

**Affiliations:** 1 Department of Psychology, University of Edinburgh, Edinburgh, UK; 2 Department of Child and Adolescent Psychiatry, Institute of Psychiatry, Psychology and Neuroscience, King’s College London, London, UK

**Keywords:** ADHD, internalising problems, exploratory mediation, regularised structural equation modelling

## Abstract

**Objective:**

ADHD and internalising symptoms such as anxiety and depression are known to be associated in adolescence and understanding the mechanisms linking them is important for improving mental health outcomes for adolescents with ADHD symptoms. Our objective was to examine these mechanisms.

**Method:**

In this study, we leverage a high-quality longitudinal dataset, the Millennium Cohort Study (*n* = 2,607 male, *n* = 2,791 female) to simultaneously evaluate a range of hypothesised mediating mechanisms. These include indirect effects via peer problems, conduct problems, self-esteem, injuries and accidents, relationships with parents, academic performance, risky decision-making, parental mental health, educational motivation, and general health. We used exploratory longitudinal mediation analysis with regularised structural equation modelling (regSEM) to examine 14 candidate mediators of the ADHD-internalising association across ages 11, 14 and 17.

**Results:**

Regularisation with lasso did not result in the de-selection of any of these mediators; however, only two were statistically significant.

**Conclusion:**

Results suggest there may be many mediators of small effect involved in the relation between ADHD symptoms and later internalising problems but point to self-esteem and parental mental health as priority mechanisms for further study in future causal and interventional research.

## Introduction

Young people with ADHD symptoms commonly experience internalising problems such as anxiety and depression, with rates in this population exceeding those of the general population (Baxter et al., 2013; Krone & Newcorn, 2015). For example, estimates suggest that approximately 25% of young people with ADHD also meet diagnostic criteria for an anxiety disorder (D’Agati et al., 2019) and approximately 40% experience a depressive episode before the age of 30 (Meinzer et al., 2013). Illuminating the mediators of this association is important for informing interventions to reduce their co-occurrence, especially through prevention of internalising problems secondary to ADHD symptoms. Adolescence is a critical period in this respect. It is associated with a range of challenges, including increased expectations of independence, academic and social stressors, and emotional changes that can increase the risk of internalising problems (Rapee et al., 2019). These challenges may compound pre-existing vulnerabilities present among young people with ADHD symptoms.

Many and varied factors have been proposed to mediate the links between ADHD symptoms and internalising problems. Theoretical models such as the cognitive behavioural and dual failure models propose, for example, that impairments or ‘failures’ in various life domains can lead to a negative self-concept and increased risk of depression (Eadeh et al., 2017; Roy et al., 2015). Indeed, studies have suggested that facing challenges such as social skills and peer difficulties such as peer rejection, victimisation or dislike; problems with school work; difficulties in relationships with parents, and behavioural problems (Eadeh et al., 2017; Eddy et al., 2018; Feldman et al., 2017; Gordon & Hinshaw, 2017; Humphreys et al., 2013; Powell et al., 2020; Roy et al., 2015) mediate associations between ADHD symptoms and internalising problems. Other models focus on co-occurring cognitive-behavioural features of ADHD and/or internalising problems as mediators. For example, studies have identified factors such as emotion (dys-)regulation (Antony et al., 2022; Bodalski et al., 2019; Murray, Wong, et al., 2021; Seymour et al., 2012), reward processing, self-control (Feldman et al., 2017), maladaptive cognitions and cognitive-behavioural avoidance (Knouse et al., 2013), sluggish cognitive tempo (Sevincok et al., 2020), locus of control (Ostrander & Herman, 2006) and rejection sensitivity (Bondü & Esser, 2015) as mediators of ADHD-internalising problem associations. Finally, a number of studies have identified a potential role of parenting, for example, overprotective parenting (Meyer et al., 2022) or parent behaviour management (Ostrander & Herman, 2006).

Previous studies analysing mediators have, however, often found that specific mediators individually explain only a modest proportion of the association between ADHD symptoms and internalising problems (Powell et al., 2020; Roy et al., 2015). This suggests that there may be a large number of mediators each of small effect. However, because existing studies have typically focused on only a handful at a time, a comprehensive picture of their combined effects remains lacking. Further, a focus on only a handful of mediators at a time makes it difficult to infer which mediating mechanisms might be of particular importance, explaining larger unique proportions of the ADHD symptoms and internalising problems association.

More comprehensive analyses can be used to build and test more encompassing theories of the relations between ADHD and internalising problems in adolescence. They can also play an important role in the early stages of designing a research programme to illuminate intervention targets. For example, comprehensive mediation analyses in existing observational data can highlight which mediators are most promising to investigate further as intervention targets. However, even when a candidate mediator is identified, confirming a causal role is challenging and cannot typically be done conclusively without resource intensive research designs (such as randomised controlled experiments). There is thus considerable value in providing initial exploratory evidence on which mediators represent promising targets for further study before embarking on confirmatory research (Serang & Jacobucci, 2020).

Exploratory mediation within a SEM framework that implements regularisation on the mediation parameters (a technique also known as ‘regSEM’) can provide this kind of evidence. It facilitates the analysis of large numbers of mediators and uses regularisation to select only those that might be considered to have non-trivial effects. The approach has been used in several previous psychosocial health studies to facilitate mediator selection where there are a large number of candidate mediators (Ammerman et al., 2018; O’Loughlin et al., 2021). For example, Ammerman et al. (2018) used regSEM to identify a subset of 11 of 46 variables that contributed to the effects of childhood maltreatment on suicidality in females, while none of the 46 candidate variables were selected in males (Ammerman et al., 2018). Similarly, Casini et al. (2021) used regSEM to select emotion regulation mediators of the association between rejection sensitivity and the outcomes of aggression, withdrawal and prosociality (Casini et al., 2022).

These applications illustrate the value of regSEM for identifying candidate mediators for further study. In this study, we, therefore, apply the technique to identify the most promising mediators in the association between ADHD symptoms and internalising problems in adolescence in a large UK-based study with adolescent participants. The goal is not to examine which mediators are causal at this stage but to identify – using large-scale observational data – a subset of mediators that may be most relevant to further examine with more causally-tailored designs in future research (e.g., in causal mediation adjusting for confounders or interventional designs). Based on data availability and past literature we evaluated a range of candidate mediators for inclusion in our models. We use longitudinal mediation analysis given the empirical importance of modelling autoregressive effects and the conceptual centrality of temporal ordering in modelling mediating effects (Maxwell & Cole, 2007). We conducted whole-sample analyses as well as analyses stratified by sex, to explore the possibility that males and females may show different patterns of mediation in the relation between ADHD symptoms and internalising problems. This is motivated by evidence that females both with and without ADHD symptoms overall show more internalising problems (Hollingdale et al., 2023; Williamson & Johnston, 2015), Recent evidence has suggested an escalation in internalising problems in the transition to adolescence that is not observed in males (Wang et al., 2026), raising the possibility that there may also be mediating mechanisms that are more salient in females than males.

## Method

### Participants

We used data from the waves of Millennium Cohort Study (MCS) (Connelly & Platt, 2014) taking place during participants’ adolescence (ages 11, 14 and 17). MCS is a UK-based longitudinal birth cohort study fully documented and available at: https://ukdataservice.ac.uk/. In brief, the first wave of MCS took place in 2000 to 2002 when participants were aged 9 months old. Participants were sampled using a stratified random sampling design in which individuals were clustered geographically and disproportionately sampled from the three smaller nations of the UK (Scotland, Wales and Northern Ireland), disadvantaged areas and ethnic minorities. For our main analyses, we used data from *n* = 5,398 participants (2,610 male, 2,788 female), reflecting the number of participants with complete data on the measures included in the current analysis. For additional analyses, the specific sample sizes are shown in the table notes. The sociodemographic information for the included subsample is provided in Table 1. To examine the impact of this selection on the representativeness of our sub-sample, we compared our sub-sample to the non-selected participants on the basis of sociodemographic variables, ADHD diagnosis and symptoms and internalising problems (see Supplementary Materials) and found that there were differences between the analytic and excluded sample on child ethnicity, parental highest academic qualifications and ADHD and internalising symptoms. Given this, where possible, sensitivity analyses including the whole sample with at least some data on the endogenous variable (*n* = 11,723), using FIML to address missing data are included.

**Table 1.** Sample Demographic Characteristics for Included Versus Excluded Cases.

Variable	Mean/category Ns	*SD*	Mean/category Ns	*SD*	*p*
	Included cases		Excluded cases		
Sex	Male = 2,607 Female = 2,791	—	Male = 4,071 Female = 4,100	—	.101
Ethnicity	White = 4,683 Mixed = 142 Indian = 100 Pakistani and Bangladeshi = 175 Black or Black British = 93 Other ethnic group = 41	—	White = 6,558 Mixed = 190 Indian = 241 Pakistani and Bangladeshi = 581 Black or Black British = 207 Other ethnic group = 130	—	<.001
Parental highest academic qualification at baseline	Higher degree = 264 First degree = 1,076 Diplomas in higher education = 587 A/AS/S levels = 599 O level/GCSE grades A-C = 1,711 GCSE grades D-G = 445 Other academic qualifications = 100 None of these qualifications = 454	—	Higher degree = 390 First degree = 1,518 Diplomas in higher education = 796 A/AS/S levels = 858 O level/GCSE grades A-C = 2,423 GCSE grades D-G = 631 Other academic qualifications = 244 None of these qualifications = 1,050	—	<.001
Family income bracket at baseline	£0 to less than £3,100 pa = 55 £3,100 to less than £10,400 pa = 745 £10,400 to less than £20,800 pa = 1,501 £20,800 to less than £31,200 pa = 1,225 £31,200 to less than £52,000 pa = 973 £52,000 and above pa = 385 Don’t Know = 260 Refused = 96	—	£0 to less than £3,100 pa = 103 £3,100 to less than £10,400 pa = 1,114 £10,400 to less than £20,800 pa = 2,228 £20,800 to less than £31,200 pa = 1,796 £31,200 to less than £52,000 pa = 1,463 £52,000 and above pa = 586 Don’t Know = 440 Refused = 188	—	.247
ADHD diagnosis (by age 11)	Diagnosed = 42 Not diagnosed = 5,312	—	Diagnosed = 83 Not diagnosed = 7,984	—	.178
ADHD symptoms age 11	2.97	2.35	2.93	2.42	<.001
Internalising symptoms age 11	1.70	1.90	1.78	1.95	.027

*Note*. *p = p*-value for a test of the difference between the included and excluded sample based on a *t*-test for continuous variables and chi-square test for categorical variables.

### Measures

#### ADHD Symptoms

ADHD symptoms were measured with the hyperactivity/inattention subscale of the Strengths and Difficulties Questionnaire (SDQ) (Goodman, 1997). The SDQ is one of the most widely used and well-validated behavioural screening instruments for children (Kersten et al., 2016), including in the current sample where it has shown good psychometric properties, including a high degree of gender, informant and developmental invariance (Murray et al., 2022; Murray, Speyer, et al., 2021). Though there is some debate around the appropriate cut-point, the hyperactivity/inattention subscale has shown good discrimination with respect ADHD diagnosis (Algorta et al., 2016; Riglin et al., 2016). The subscale includes five items that refer to the last six months, with reference to the following behaviours: ‘restless, overactive, cannot stay still for long’; ‘constantly fidgeting or squirming’; ‘easily distracted, concentration wanders’; ‘thinks things out before acting’; and ‘sees tasks through to the end, good attention span’. Responses were provided on a 3-point Likert-type scale with options: *not true* (0), *somewhat true* (1) and *certainly true* (2). Positively worded items were reverse-coded, and item responses were summed to produce an overall hyperactive/inattentive score with higher scores indicating greater hyperactivity/inattentiveness (possible range = 0–10). We used the parent-reported version of the SDQ, as the only version available at all of age 11, 14 and 17.

#### Internalising Problems

Internalising symptoms were measured with the emotional problems subscale of the SDQ (described above). The emotional problems items refer to: *often complaining of headaches, stomach-aches or sickness; having many worries; being often unhappy, down-hearted, or tearful; being nervous or clingy in new situations; and having many fears, being easily scared*.

#### Candidate Mediators

Full details of the candidate mediator measures are provided in Supplementary Materials. They were included if (i) they were available at both age 11 and age 14, in order to allow for the adjustment of relevant autoregressive effects in the longitudinal mediation model; (ii) prior theory or empirical studies have suggested they could plausibly act as a mediator in the association between ADHD symptoms and internalising problems in adolescence. They were: prosociality, peer problems, conduct problems, self-esteem, injuries and accidents, closeness of relationship with primary caregiver, academic performance, risk-taking behaviours on the Cambridge Gambling Task (CGT), parental mental health and educational motivation.

### Statistical Procedure

#### Exploratory Mediation Analysis

We followed the exploratory mediation analysis with SEM procedure described by Serang et al. (2017), modifying it for longitudinal analysis. We first specified a full SEM model in which age 17 internalising problems were regressed on age 14 mediating variables and age 11 ADHD symptoms (the ‘direct’ effect) and age 14 mediating variables were regressed on age 11 ADHD symptoms. The 14 indirect effects were calculated as the products of the coefficients for the regression of internalising problems (age 17) on the relevant mediator (age 14) and the regression of that mediator on ADHD symptoms (age 11). We additionally included the regression of age 17 internalising problems on age 14 internalising problems and ADHD symptoms and the autoregressive and cross-lagged effects of ADHD symptoms, internalising problems and mediators across the age 11 to age 14 lag. We did not include the mediators at age 17 given our focus on their role as mediators and because not all mediators were available at age 17. Where relevant, latent variable measurement models for constructs were used (i.e., for ADHD symptoms, internalising problems, prosociality, peer problems, conduct problems, self-esteem and parental mental health). The full model specification can be seen in the R code: https://osf.io/ap95r

**Figure 1.**
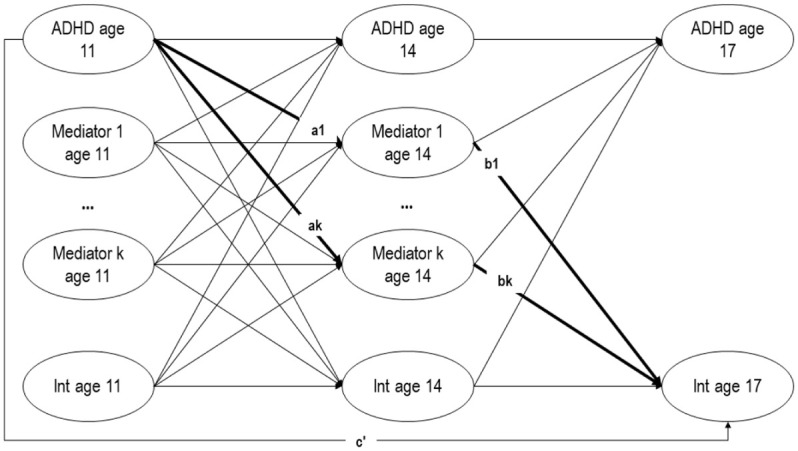
Simplified path mediation model.

Figure 1 provides a simplified example of the multiple path mediation model fitted. Paths a1-ak label the paths from ADHD symptoms to the mediators, paths b1-bk label the paths from the mediators to internalising problems, and c′ is the direct effect from ADHD symptoms to internalising problems. From the a and b parameters, indirect effects via each mediator (shown in bold) are derived by finding the product a*b. The total effect of ADHD symptoms on internalising problems is then the sum of the indirect effects and c′.

In the approach proposed by Serang et al. (2017), the above-described multiple mediation model is fit using regularised SEM (‘RegSEM’) (Jacobucci et al., 2016). Regularised SEM incorporates a penalty term into the maximum likelihood function used for model fitting:

(1) $$\begin{array}{c} F=\log\left(\left|\Sigma \right|\right)+tr\left(C*\Sigma^{-1}\right)-\log\left(\left|C\right|\right)\\ -p+\lambda P\left(.\right) \end{array}$$

where Σ is the expected covariance matrix, *C* is the observed covariance matrix, *p* is the total number of manifest variables, λ is a tuning parameter and P(.) is a function for summing over the absolute value of the penalised coefficients. In exploratory mediation applications of SEM this is the a and b coefficients. We used a general purpose optimiser (rsolnp) which provides an approximate solution and thresholding to set parameters very close to 0 to 0 (Orzek et al., 2023). This was implemented within the regsem R package (Li et al., 2021).

First, all continuous variables were standardised to have a mean of 0 and variance of 1 (for the constructs specified using latent variables the latent variable variance was fixed to 1). Next, the tuning parameter value was found by testing 100 λ values (0–1 in 0.01 increments) and identifying the model with the smallest Bayesian Information Criterion (BIC). BIC provides a more practical solution to tuning by cross-validation, which can become impractical for large models due to the computation time involved. The model was then re-fit without regularisation, using only the subset of mediators with non-zero indirect effects from the model with the optimal λ value. Statistical significance of the indirect effects was assessed using the delta method given that the computation time involved in using bootstrapped standard errors for this model would be prohibitively long. Previous results have suggested that for our sample size, this should not substantively impact results (Hayes & Scharkow, 2013). Further, after checking that it did not substantially impact results compared to FIML in a full (non-regularised) model, complete case analysis was used based on pragmatic considerations given the computation time that would be needed to utilise FIML or multiple imputation with tuning. If there were substantive differences between the initial complete case and FIML results, additional sensitivity analyses were considered.

Results from the final models are reported in terms of the direct and indirect effects, with the fully standardised indirect effect used as an effect size measure. The fully standardised indirect effect is here defined as:

(2) $$ab_{cs}=ab\frac{\sigma_{X}}{\sigma_{Y}}$$

This is equivalent to the product of standardised regression coefficients and is recommended over measures such as the ‘proportion of mediated effect’ because it has fewer limitations/ more desirable properties as an effect size metric (Lachowicz, 2017). It can be interpreted in a similar way to the standardised regression coefficient and represents the expected standard deviation (SD) change in the outcome variable (internalising problems) for a one SD increase in the predictor (ADHD symptoms) via the relevant mediator variable.

Analyses were first run on the whole sample, followed by male and female subsamples, the latter to explore the possibility that there are different mediating mechanisms in males versus females.

## Results

### Descriptive Statistics

Descriptive statistics are provided in Table S1 of Supplementary Materials.

### Initial Models with No Regularisation in Whole Sample

R code is provided at: https://osf.io/ap95r. For reference, SEMs fit without any regularisation are provided in Table 2 (complete case analysis) and Table 3 (FIML analysis). The complete case analysis yielded three significant mediators: self-esteem (β = −.092, *p* < .001), risk adjustment (β = −.009, *p* = .034) and parental mental health (β = −.02, *p* = .002). When fitting this model with FIML to utilise the whole sample (*n* = 11,723), the pattern of results was very similar, with significant mediating effects for self-esteem and parental mental health. Risk adjustment, which was on the margins of statistical significance in the complete case analysis was not significant in the FIML analysis.

**Table 2.** Indirect Effects Prior to Regularisation for Complete Case Analysis in Whole Sample.

Mediator	*B*	*SE*	z	*p*	β
Prosociality	−0.007	0.031	−0.242	.808	−.005
Peer problems	−0.002	0.026	−0.069	.945	−.001
Conduct problems	−0.041	0.052	−0.794	.427	−.027
Self-esteem	−0.092	0.026	−3.57	<.001*	−.061
Accident and injuries	0.000	0.000	0.209	.835	.000
Parental closeness	0.005	0.010	0.539	.590	.003
Academic performance	0.002	0.009	0.183	.855	.001
Risk-taking	−0.001	0.005	−0.141	.888	.000
Risk decision-making quality	0.004	0.003	1.263	.207	.003
Risk deliberation time	0.004	0.003	1.496	.135	.003
Risk adjustment	−0.009	0.004	−2.121	.034*	−.006
Delay aversion	−0.003	0.003	−1.178	.239	−.002
Parental mental health	−0.02	0.006	−3.029	.002*	−.013
Educational motivation	0.003	0.021	0.117	.907	.002

*Note*. Analysis includes the *n* = 5,398 with complete data on all analytic variables. *B* = indirect effect via mediator; β = standardised indirect effect; SE = standard error.

* significant at *p* < .05.

**Table 3.** Indirect Effects Prior to Regularisation Using FIML in Whole Sample.

Mediator	*B*	*SE*	*z*	*p*	β
Prosociality	.008	0.02	0.431	.667	.006
Peer problems	−.013	0.018	−0.703	.482	−.009
Conduct problems	−.051	0.035	−1.466	.143	−.034
Self-esteem	−.099	0.024	−4.119	<.001*	−.066
Accident and injuries	0.000	0.000	0.354	.723	.000
Parental closeness	.006	0.007	0.911	.362	.004
Academic performance	−.003	0.008	−0.365	.715	−.002
Risk-taking	−.005	0.003	−1.572	.116	−.003
Risk decision-making quality	0.000	0.002	−0.18	.857	.000
Risk deliberation time	.004	0.002	1.665	.096	.003
Risk adjustment	−.003	0.003	−0.996	.319	−.002
Delay aversion	0.000	0.001	−0.209	.835	.000
Parental mental health	−.014	0.005	−2.96	.003*	−.009
Educational motivation	.012	0.02	0.61	.542	.008

*Note*. Analysis includes the *n* = 11,723 with data on the endogenous variables. *B* = indirect effect via mediator; β = standardised indirect effect; SE = standard error.

* Significant at *p* < .05.

### Regularised Models in Whole Sample

After discarding any models with tuning parameters that did not converge (which included all models with a λ > .40 and some models below this value), the tuning phase yielded an optimal λ of 0. Applying this (lack of) penalty indicated no mediators for de-selection (i.e., none of the corresponding coefficients were driven to 0) and the final model, therefore, included all mediators. The indirect effect estimates are thus simply those in Tables 2 and 3. The direct effect of ADHD symptoms on later internalising problems in the complete case analysis was *B* = .177 (*p* = .004). In the FIML analysis, the direct effect was *B* = .179 (*p* = .002). Despite the selection of all mediators, only two: self-esteem and parental mental health had statistically significant mediating effects in the FIML analysis and a third (risk adjustment) was significant in the complete case analysis. These results indicate that increases in ADHD symptoms result in increased internalising problems via decreased self-esteem and poorer parental mental health. The effect via risk adjustment can be considered less robust since it appears only in the complete case analyses (which may be affected by non-random attrition) and was less statistically significant than the other mediators in those analyses. Nevertheless, it can be interpreted as increases in ADHD symptoms resulting in increased internalising problems via a tendency to bet higher on high-probability compared to low-probability trials on a gambling task.

#### Initial Models with no Regularisation in Male and Female Subsamples

The indirect effects from sex-stratified SEMs without regularisation are provided in Tables 4 and 5 (complete case and FIML analyses respectively). Following the analyses in the whole sample that suggested that any λ values >.40 were not likely to be viable, we focused on λ up to a maximum of only .40 in the sex-stratified analyses. Prior to regularisation (i.e., in a model including all candidate mediators), risk-taking was the only significant mediator in the male sub-sample (*B* = 0.017, *p* = .037) alongside a direct effect of *B* = 0.177 (*p* < .004). This indirect effect can be interpreted as ADHD symptoms increasing internalising problems via an increased tendency to greater reward sensitivity/lower punishment sensitivity. In the male sub-sample FIML analyses including all mediators, the direct effect of ADHD symptoms on internalising problems was *B* = 0.254 (*p* = .003); however, there were no significant indirect effects.

**Table 4.** Indirect Effects Prior to Regularisation Using Complete Case Analysis in Male and Female Sub-samples.

Mediator	*B*	*SE*	*z*	*p*	β	*B*	*SE*	*z*	*p*	β
	Male sub-sample					Female sub-sample				
Prosociality	−0.059	0.055	−1.072	.284	−.04	−0.007	0.046	−0.157	.875	−.005
Peer problems	−0.022	0.043	−0.506	.613	−.015	−0.055	0.038	−1.458	.145	−.037
Conduct problems	0.057	0.116	0.490	.624	.039	−0.002	0.06	−0.039	.969	−.002
Self-esteem	−0.013	0.018	−0.691	.490	−.009	−0.078	0.038	−2.036	.042*	−.053
Accident and injuries	−0.002	0.003	−0.782	.434	−.002	0.000	0.001	−0.075	.94	.000
Parental closeness	0.002	0.014	0.132	.895	.001	0.005	0.014	0.385	.700	.004
Academic performance	0.005	0.005	1.006	.315	.004	0.000	0.014	−0.001	.999	.000
Risk-taking	0.017	0.008	2.083	.037*	.012	0.003	0.004	0.682	.495	.002
Risk decision-making quality	0.004	0.007	0.585	.558	.003	−0.005	0.005	−0.886	.376	−.003
Risk deliberation time	−0.006	0.005	−1.216	.224	−.004	0.009	0.005	1.615	.106	.006
Risk adjustment	−0.011	0.008	−1.353	.176	−.008	0.002	0.004	0.438	.661	.001
Delay aversion	−0.004	0.008	−0.483	.629	−.003	0.000	0.002	−0.155	.877	.000
Parental mental health	−0.016	0.009	−1.804	.071	−.011	−0.027	0.011	−2.445	.014*	−.018
Educational motivation	−0.014	0.017	−0.813	.416	−.009	0.014	0.034	0.413	.679	.009

*Note*. Analysis includes the *n* = 2,609 with complete data on all analytic variables. *B* = indirect effect via mediator; β = standardised indirect effect; SE = standard error.

* Significant at *p* < .05.

**Table 5.** Indirect Effects Prior to Regularisation Using FIML in Male and Female Sub-samples.

Mediator	*B*	*SE*	*z*	*p*	β	*B*	*SE*	*z*	*p*	β
	Male sub-sample					Female sub-sample				
Prosociality	−0.015	0.031	−0.474	0.636	−.01	0.005	0.042	0.126	.900	.004
Peer problems	−0.025	0.031	−0.813	0.416	−.017	−0.081	0.037	−2.195	.028*	−.055
Conduct problems	−0.006	0.061	−0.093	0.926	−.004	−0.033	0.06	−0.551	.581	−.023
Self-esteem	−0.008	0.019	−0.442	0.658	−.006	−0.117	0.045	−2.564	.010*	−.079
Accident and injuries	−0.003	0.003	−1.329	0.184	−.002	0	0	0.121	.904	0
Parental closeness	0.001	0.01	0.132	0.895	.001	0.013	0.014	0.909	.363	.009
Academic performance	0.005	0.008	0.586	0.558	.003	−0.004	0.016	−0.251	.802	−.003
Risk-taking	0.009	0.005	1.893	0.058	.006	0.002	0.003	0.811	.418	.001
Risk decision-making quality	0	0.001	0.197	0.844	0	−0.004	0.004	−0.959	.338	−.002
Risk deliberation time	0.002	0.002	0.854	0.393	.001	0.008	0.005	1.504	.133	.005
Risk adjustment	−0.005	0.004	−1.268	0.205	−.004	0.004	0.004	1.032	.302	.003
Delay aversion	0.007	0.005	1.302	0.193	.005	0	0.002	−0.231	.817	0
Parental mental health	−0.008	0.006	−1.349	0.177	−.005	−0.027	0.01	−2.695	.007*	−.018
Educational motivation	−0.012	0.019	−0.643	0.520	−.008	0.037	0.04	0.934	.350	.025

*Note*. Analysis includes the *n* = 5,696 with data on the endogenous variables. *B* = indirect effect via mediator; β = standardised indirect effect; SE = standard error.

* Significant at *p* < .05.

In the female sub-sample, the significant mediating effects via self-esteem (*B* = 0.078, *p* = .042) and parental mental health (*B* = −0.027, *p* = .014) seen in the whole-sample analyses were present but there were no other significant mediating effects. The direct effect was non-significant (*B* = 0.093; *p* = 230). In the female sub-sample, FIML analyses the direct effect of ADHD symptoms on internalising problems was also non-significant (*B* = 0.114, *p* = .092) and there were three significant mediators: peer problems (*B* = −0.081, *p* = .028), self-esteem (*B* = −0.117, *p* = .010) and parental mental health (*B* = −0.027, *p* = .007).

#### Regularised Models in the Male and Female Subsamples

Tuning in the male sub-sample yielded an optimal λ = 0.03 for the complete case analyses. No models converged with values >.10. Adopting λ = 0.03 value resulted in the de-selection of all but two mediators (risk-taking deliberation time and risk adjustment. Re-fitting the model with only these two mediating effects yielded the final complete case analysis for males, provided in Table 6. Despite both being selected by lasso, only the deliberation time effect was statistically significant. This can be interpreted as ADHD symptoms increasing internalising problems via increased deliberation time in risky decision making. The direct effect in this model was 0.276 (*p* = .005). We also fit this final model using FIML (Table 7), which in which both indirect effects were significant. The second significant indirect effect in this model can be interpreted as an increase in internalising problems due to ADHD symptoms via a tendency to bet higher on high-probability compared to low-probability trials on a gambling task.

**Table 6.** Indirect Effects for the Final Regularised Models Using Complete Case Analysis in the Male and Female Subsamples.

Mediator	*B*	*SE*	*z*	*p*	β	*B*	*SE*	*z*	*p*	β
	Male sub-sample					Female sub-sample				
Prosociality	—	—	—	—	—	—	—	—	—	—
Peer problems	—	—	—	—	—	0.007	0.005	1.461	.144	.005
Conduct problems	—	—	—	—	—	—	—	—	—	—
Self-esteem	—	—	—	—	—	—	—	—	—	—
Accident and injuries	—	—	—	—	—	—	—	—	—	—
Parental closeness	—	—	—	—	—	—	—	—	—	—
Academic performance	—	—	—	—	—	.015	.006	2.539	.011*	.010
Risk-taking	—	—	—	—	—	—	—	—	—	—
Risk decision-making quality	—	—	—	—	—	—	—	—	—	—
Risk deliberation time	0.010	0.003	2.816	.005*	.007	—	—	—	—	—
Risk adjustment	0.004	0.004	0.911	.362	.003	—	—	—	—	—
Delay aversion	—	—	—	—	—	—	—	—	—	—
Parental mental health	—	—	—	—	—	—	—	—	—	—
Educational motivation	—	—	—	—	—	—	—	—	—	—

*Note*. Analysis includes the *n* = 3,373 males and *n* = 3,616 females with complete case data on the included variables. *B* = indirect effect via mediator; β = standardised indirect effect; SE = standard error.

* Significant at *p* < .05.

**Table 7.** Indirect Effects for the Final Regularised Models Using FIML in the Male and Female Subsamples.

Mediator	*B*	*SE*	*z*	*p*	β	*B*	*SE*	*z*	*p*	β
	Male sub-sample					Female sub-sample				
Prosociality	—	—	—	—	—	—	—	—	—	—
Peer problems	—	—	—	—	—	0.008	.004	2.049	.040*	.006
Conduct problems	—	—	—	—	—	—	—	—	—	—
Self-esteem	—	—	—	—	—	—	—	—	—	—
Accident and injuries	—	—	—	—	—	—	—	—	—	—
Parental closeness	—	—	—	—	—	—	—	—	—	—
Academic performance	—	—	—	—	—	0.014	0.005	2.632	.008*	.010
Risk-taking	—	—	—	—	—	—	—	—	—	—
Risk decision-making quality	—	—	—	—	—	—	—	—	—	—
Risk deliberation time	0.008	0.003	2.723	.006*	.006	—	—	—	—	—
Risk adjustment	0.006	0.004	1.574	.004*	.004	—	—	—	—	—
Delay aversion	—	—	—	—	—	—	—	—	—	—
Parental mental health	—	—	—	—	—	—	—	—	—	—
Educational motivation	—	—	—	—	—	—	—	—	—	—

*Note*. Analysis includes the *n* = 6,360 males and *n* = 6,638 females with data on the included variables. *B* = indirect effect via mediator; β = standardised indirect effect; SE = standard error.

* Significant at *p* < .05.

In the female sub-sample, the tuning phase yielded an optimal λ = 0.05. This resulted in the de-selection of all but two mediators: peer problems and academic performance. Re-fitting the model with only these two mediating effects gave the final model shown in Table 6. However, only one of these: self-reported academic performance was statistically significant. This effect can be interpreted as ADHD symptoms increasing internalising problems via poorer academic performance. The direct effect from this model was non-significant (*B* = −0.041, *p* = .578). When re-fitting the model with FIML, both mediating effects were statistically significant. The additional peer problems effect in this model can be interpreted as ADHD symptoms resulting in higher internalising problems via increased peer problems. The direct effect in that model was non-significant (*B* = 0.018, *p* = .801).

## Discussion

The present study provided an exploratory analysis of 14 candidate mediators in the association between ADHD and internalising symptoms across adolescence. Lasso within regularised SEM selected all candidate mediators in whole sample analyses; however, only two (self-esteem and parental mental health) were statistically significant in both the FIML and complete case analysis variants. This pattern of findings (with standardised indirect effects all < |.10|) is consistent with the idea that there may be many mediators of small effect contributing to the links between ADHD symptoms and later internalising problems in adolescence. Self-esteem and parental mental health: the only two robustly significant mediators, are indicated as priority targets for further study in interventional research. However, there were also some indications of sex differences in mechanisms which merit exploration in future studies. Specifically, in male adolescents, risk-taking tendencies on a gambling task tended to be selected by lasso and/or statistically significant, whereas in female adolescents, self-esteem, parental mental health and peer problems were selected or significant across the models.

The whole-sample findings of the current study imply that focusing on just a handful of mediators to address the links between ADHD symptoms and internalising problems will provide only a limited piece of the full picture. However, from a practical perspective there are difficulties in measuring and modelling large numbers of mediators simultaneously. Space is at a premium in data collections and researchers will typically need to keep the number of measures as few as possible to minimise participant burden, especially for young people with ADHD or ADHD symptoms (Murray et al., 2023). In providing a means to apply principled variable selection to large models, regularised SEM in pre-existing high quality data may help to optimise the use of limited space in studies by pointing to the most promising mediators to measure (Serang et al., 2017). By providing preliminary indications of which candidate mediators may be involved in the ADHD-internalising problems association, our findings can help focus the resources of more rigorous but resource intensive research designs on mediators that are likely to be the most promising. This includes longitudinal research with suitable controls for both time-invariant and time-varying confounding, causal mediation analyses, or experimental designs, which are typically expensive and logistically challenging to implement (Imai et al., 2010; Keogh et al., 2018; Streeter et al., 2017).

If the findings of the present study are confirmed by these designs, they would suggest that interventions to reduce the risk of developing co-occurring internalising problems among adolescents with ADHD may be most effective if they address multiple mechanisms. These may include cognitive-behavioural features associated with ADHD (e.g., risk-taking tendencies), socio-environmental stressors (e.g., school and peer problems) and their impacts (e.g., self-esteem). However, some mediators appeared to be particularly key based on the present analysis. In particular, self-esteem and parental mental health emerged as having the largest unique effects. These findings are consistent with developmental cascade models which propose that difficulties associated with ADHD can undermine young people’s self-esteem and lead to an increased risk of anxiety and depression (Roy et al., 2015). It is also consistent with previous research, including that in the current sample which found that ADHD symptoms relate to the development of lower self-esteem in adolescence (Russell et al., 2025). Taken together, these findings suggest benefits to addressing self-esteem as an intervention target among adolescents with ADHD symptoms. Whilst effective interventions for self-esteem exist, further work is needed to adapt/tailor and evaluate their effects specifically for adolescents with ADHD symptoms (Niveau et al., 2021), including their impact on internalising problems.

The mediating effects of parental mental health are also consistent with previous findings suggesting that adolescent ADHD symptoms are related to poorer parental mental health (Ko & Jeong, 2024) which has in turn been linked to young person mental health (Speyer et al., 2022). Such findings are consistent with the idea that whole family system interventions (Lagdon et al., 2021; Moltrecht et al., 2024) may be an optimal approach for families with members experiencing ADHD symptoms. Similarly, screening parents of adolescents with ADHD symptoms for mental health symptoms to facilitate intervention provision could help contribute to the prevention of adolescent mental health symptoms.

However, our findings also suggested that different mechanisms may be relatively more/less important in male versus female adolescents. Overall, the findings for females largely corresponded to the ‘dual failure’ model in which difficulties in the academic and social domains which impact self-concept and ultimately engender internalising problems (Roy et al., 2015). Yet for males, it was the risk-decision making variables that tended to be selected or significant across the models. While ADHD-related differences in risk-taking behaviour and strategies have been observed in previous research (Dekkers et al., 2020), their role in internalising problems remains to be clarified.

Other of our findings on sex differences are consistent with previous research. The fact that peer problems emerged as a significant mediator in females only aligns with the proposal that the peer problems associated with ADHD may be more impactful for females because they place greater importance on and tend to have more intimate peer networks characterised by higher levels of peer attachment than males (Kok et al., 2016). Consistent with this, one previous longitudinal study found a significant mediating effect of peer problems in the relation between ADHD symptoms and depression (7% mediation for peer dislike and 3% mediation for victimisation) in females but not males (Roy et al., 2015). The finding of a mediating effect of parental mental health in females only, is similarly consistent with previous analyses suggesting that female adolescent internalising symptom may be more closely related to parental mental health than male’s (Zhu et al., 2023). Finally, the finding of a mediating effect of self-esteem in females only is consistent with previous research suggesting that low self-esteem may be more prominent in girls compared to boys with ADHD (Kok et al., 2016). The consistency of these findings with previous theory and empirical evidence helps to bolster the idea that they are priority mediating mechanisms for further study in female adolescents with ADHD.

### Limitations

It is important to consider the limitations of the present study. First, not all possible mediators that merit exploration were included due to data availability. Mediators such as emotion dysregulation, sluggish cognitive tempo, rumination, and executive functions are also likely to play a mediating role and would be valuable to include in future comprehensive mediation analyses (Broletti et al., 2024; Kandeğer et al., 2023). The idea that the inclusion of mediators was incomplete in the present study is also consistent with the significant direct effect in the whole-sample and male-specific analyses that remained after including the 14 indirect effects. There were also some measurement limitations in the mediators, for example, some were measured with a single item that could not capture the complexity of the concepts involved. Further, the measures of ADHD symptoms and internalising problems in the sample do not offer separate subscales for different symptom domains, therefore, it is not possible to distinguish mediators in the relations between inattention versus hyperactivity/impulsivity and anxiety versus depression symptoms. As regards the representativeness of the sample, although MCS was collected according to a complex survey design, design-adjusted models (incorporating weighting, stratification and clustering variables) are not readily implementable in the context of regularised SEM. As such, the analyses were not design-adjusted and may therefore be limited in their generalisability to the underlying UK population. Similarly due to the computation time involved in the tuning phase, we used complete case analysis for this; however, we did fit all initial and final models with FIML as well to check there were no major discrepancies indicative of strong bias in the complete case analyses. Finally, we did not have a sufficiently large subsample of adolescents diagnosed with ADHD to conduct meaningful subgroup analyses within this group only. Extending the present research to clinically diagnosed samples would be a valuable future direction to establish the extent to which the same mediators emerge as significant among those with ADHD.

## Conclusions

Based on longitudinal mediation analyses with lasso-based variable selection there are likely to be a large number of mediators with small effects responsible for linking ADHD symptoms to internalising problems. Key mediators were self-esteem and parental mental health and these represent priority mediators for further study in interventional research. There was also evidence for different mechanisms in males versus females, with risk-taking variables of key importance in the former and academic and social difficulties of key relevance in the latter.

## Supplemental Material

sj-docx-1-jad-10.1177_10870547261419589 – Supplemental material for Identifying Candidate Mediators Linking ADHD Symptoms and Internalising Problems in Adolescence: An Exploratory Longitudinal Mediation Analysis Supplemental material, sj-docx-1-jad-10.1177_10870547261419589 for Identifying Candidate Mediators Linking ADHD Symptoms and Internalising Problems in Adolescence: An Exploratory Longitudinal Mediation Analysis by Aja Louise Murray, Katie Dryburgh and Edmund J. S. Sonuga-Barke in Journal of Attention Disorders
