# Supplementary material for: Identifying Candidate Mediators Linking ADHD Symptoms and Internalising Problems in Adolescence: An Exploratory Longitudinal Mediation Analysis
Source: J Atten Disord. 2026 Mar 6;30(8):986–98. doi: 10.1177/10870547261419589 (PMC13328922; doi:10.1177/10870547261419589)
Supplement: sj-docx-1-jad-10.1177_10870547261419589 – Supplemental material for Identifying Candidate Mediators Linking ADHD Symptoms and Internalising Problems in Adolescence: An Exploratory Longitudinal Mediation Analysis [file sj-docx-1-jad-10.1177_10870547261419589.docx]

**Supplementary Materials**

**Table S1: Descriptive statistics for study variables**

| **Study variable** | **N** | **Mean** | **SD** |
| --- | --- | --- | --- |
| Age 11 ADHD symptoms | 5395 | 2.78 | 2.35 |
| Age 11 emotional symptoms | 5397 | 1.70 | 1.90 |
| Age 11 prosociality | 5397 | 8.90 | 1.45 |
| Age 11 peer problems | 5397 | 1.19 | 1.56 |
| Age 11 conduct problems | 5397 | 1.19 | 1.43 |
| Age 11 self-esteem | 5395 | 8.01 | 2.13 |
| Age 11 accidents and injuries | 5398 | 0.54 | 0.95 |
| Age 11 parental closeness | 5398 | 3.53 | 0.61 |
| Age 11 academic performance | 5397 | 5.51 | 1.43 |
| Age 11 CGT risk-taking | 5395 | 0.52 | 0.17 |
| Age 11 CGT quality of decision making | 5395 | 0.82 | 0.16 |
| Age 11 CGT deliberation time | 5395 | 3302.79 | 1291.63 |
| Age 11 CGT risk adjustment | 5395 | 0.76 | 1.05 |
| Age 11 CGT delay aversion | 5395 | 0.28 | 0.24 |
| Age 11 parental mental health | 5398 | 26.41 | 4.01 |
| Age 11 academic motivation | 5396 | 16.13 | 2.29 |
| Age 14 ADHD symptoms | 5398 | 2.64 | 2.30 |
| Age 14 emotional symptoms | 5398 | 1.85 | 2.03 |
| Age 14 prosociality | 5398 | 8.45 | 1.73 |
| Age 14 peer problems | 5398 | 1.55 | 1.71 |
| Age 14 conduct problems | 5398 | 1.22 | 1.48 |
| Age 14 self-esteem | 5396 | 9.40 | 2.90 |
| Age 14 accidents and injuries | 5398 | 0.60 | 1.18 |
| Age 14 parental closeness | 5398 | 3.34 | 0.70 |
| Age 14 academic performance | 5397 | 9.15 | 1.69 |
| Age 14 CGT risk-taking | 5396 | 0.51 | 0.15 |
| Age 14 CGT quality of decision making | 5396 | 0.90 | 0.12 |
| Age 14 CGT deliberation time | 5396 | 2269.79 | 860.02 |
| Age 14 CGT risk adjustment | 5396 | 1.12 | 0.97 |
| Age 14 CGT delay aversion | 5396 | 0.26 | 0.20 |
| Age 14 parental mental health | 5398 | 25.96 | 3.92 |
| Age 14 academic motivation | 5396 | 14.75 | 2.42 |
| Age 17 ADHD symptoms | 5360 | 2.27 | 2.17 |
| Age 17 emotional symptoms | 5360 | 1.90 | 2.17 |

*Note.* CGT = Cambridge gambling task. Descriptive statistics are based on cases included in the complete case analyses. For measures for which latent variable models are used in the main analyses , the descriptive statistics are based on sum scores.

**Measures**

The following mediators were included in the present study. ‘mx’ refers to the mediator number within the relevant model. To be included, variables needed to be available at least at age 11 and 14 in order that the autoregressive effects of both ADHD symptoms and the mediators could be taken into account.

*Prosociality, peer problems* and *conduct problems* were measured via parent reports (m1-m3) using the strengths and difficulties questionnaire (SDQ; Goodman, 1997). Each was measured using 5 items with responses recorded on a 3-point scale. Prosociality items referred to behaviours such as sharing and helping; peer problems items to issues such as being bullied or being solitary; conduct problems referred to issues such as stealing or fighting. Unidimensional latent variable measurement models were used for each concept for each informant to provide overall scores. In the descriptive statistics, higher scores represent higher levels of each concept.

*Self-esteem* (m4) was measured with the *Rosenberg Self-Esteem Scale* (Rosenberg, 1965)*.* The version used in MCS is a five-item version that includes items such as I feel that I am a person of worth” and “I feel good about myself”. Responses were recorded on a 4-point scale from *strongly disagree* to *strongly agree.*  As all items are worded ‘positively’, higher scores (indicating stronger disagreement) represent worse self-esteem Overall scores were obtained using a unidimensional latent variable measure model. In the current sample it has shown good psychometric properties, including high internal consistency values (Carter et al., 2024; Russell et al., 2025)

*Injuries and accidents* (m5) were measured using a single item indicating the number of accidents or injuries experienced by the cohort member.

*Parental closeness* (m6) was measured using a single parent-reported item asking parents to report, overall how close they felt they were to the cohort member. Responses were recorded on a scale from *1 = not very close*  to *4* = *extremely close.*

*Self-reported academic performance* (m7) was measured using the sum of three self-report items measuring the extent to which respondents endorse being good at English, maths, and science. Responses were recorded on an X-point scale from 1= *strongly agree*  to 4 = *strongly disagree*, meaning that higher scores mean poorer self-perceived academic performance.

*Risk decision making* was measured by the Cambridge Gambling Task (CGT). In the CGT, participants are instructed to bet on the location of a token within a set of boxes displayed on a computer screen. They make their choice by selecting a box colour (red or blue) and how much to bet are presented with 10 boxes in varying proportions of red and blue colour across trials. Following practice trials to ensure they understand the task, participants begin the task with 100 points and can select between 5 and 95% of their points to bet in each trial. A slider moves between these points on the scale and participants press a button to halt the slider and select the proportion they would like to bet. The task yields sic outcome measures.

*Risk-taking* (m8) can be calculated as the mean proportion of points bet on trials where the most probable colour was selected with higher scores representing greater reward sensitivity/lower punishment sensitivity*. Quality of decision-making* (m9) is calculated as the mean proportion of trials where the most probable colour was selected. *Deliberation time* (m10) is the mean time taken in milliseconds to make a box colour selection. *Risk adjustment* (m11) is calculated based on the extent to which bets are moderated by probability and reflects the tendency to bet higher on high-probability compared to low-probability trials. *Delay aversion* (m12) is the time that participants wait are willing to wait to place a higher or lower bet. *Overall bet proportion* is the mean proportion of points gambled across all trials. In line with previous research (Hosozawa et al., 2021), this latter score was not included because of a high level of redundancy (*r*>.90) with the *risk-taking* score. The other five dimensions were used as separate scores.

*Parental mental health* (m13) was measured using the Kessler scales (K6), which measures self-reported parental symptoms such as hopelessness, restlessness, feeling worthless, and feeling nervous over the previous 30 days. Responses are recorded on an 5-point scale from *all of the time* to *none of the time,* meaning that higher scores represent better mental health. Overall scores were estimated using a unidimensional latent variable model.

*Educational motivation* (m14) was measured using five self-reported items measuring school motivation: ‘how often do you try your best at school?’, ‘how often do you find school interesting?’, ‘how often do you feel unhappy at school?’, ‘how often do you get tired at school?’ and ‘how often do you feel school is a waste of time?’. Responses are recorded on a scale from 1*= all of the time to* *3 = never.* After reverse coding the two oppositely worded items, an average score across the items was formed.

**References**

Carter, L., Speyer, L., Caye, A., Rohde, L., & Murray, A. L. (2024). Late adolescent outcomes of different developmental trajectories of ADHD symptoms in a large longitudinal study. *European Child & Adolescent Psychiatry*. https://doi.org/10.1007/s00787-024-02516-5

Goodman, R. (1997). The Strengths and Difficulties Questionnaire: A research note. *Journal of Child Psychology and Psychiatry*, *38*(5), 581–586.

Hosozawa, M., Mandy, W., Cable, N., & Flouri, E. (2021). The role of decision-making in psychological wellbeing and risky behaviours in autistic adolescents without ADHD: Longitudinal evidence from the UK millennium cohort study. *Journal of Autism and Developmental Disorders*, *51*, 3212–3223.

Rosenberg, M. (1965). Rosenberg self-esteem scale (SES). *Society and the Adolescent Self-Image*.

Rosenberg, M. (1979). Society and the Adolescent self-image: Conceiving the self. *New York: Basic Book, Inc*.

Russell, A., Hoxha, D., & Murray, A. (2025). Developmental relations between ADHD and self-esteem: Evaluating peer problems as a mediating mechanism. *European Child & Adolescent Psychiatry*. https://doi.org/10.1007/s00787-025-02752-3
